# *N*-[2,4-Dioxo-3-aza­tricyclo­[7.3.1.0^5,13^]trideca-1(13),5,7,9,11-pentaen-3-yl]thio­urea

**DOI:** 10.1107/S1600536812021812

**Published:** 2012-05-19

**Authors:** Rashad Al-Salahi, Mohamed Al-Omar, Mohamed Marzouk, Seik Weng Ng

**Affiliations:** a Department of Pharmaceutical Chemistry, College of Pharmacy, King Saud University, Riyadh 11451, Saudi Arabia; b Department of Chemistry, University of Malaya, 50603 Kuala Lumpur, Malaysia; c Chemistry Department, Faculty of Science, King Abdulaziz University, PO Box 80203 Jeddah, Saudi Arabia

## Abstract

In the two independent mol­ecules in the asymmetric unit of the title compound, C_13_H_9_N_3_O_2_S, the aza­tricyclo­trideca­penta­ene ring system is approximately planar with r.m.s. deviations of 0.022 and 0.033 Å. The urea unit connected to the fused rings is approximately perpendicular [dihedral angles = 82.4 (1) and 82.7 (1)°]. In the crystal, the mol­ecules associate by N—H⋯O hydrogen bonds, forming a chain running along the *a* axis. The crystal studied was a non-merohedral twin with a fractional contribution of 49.6 (1)% for the minor domain.

## Related literature

For background to the class of anti­tumor drugs, see; Pessoa *et al.* (2010 ▶).
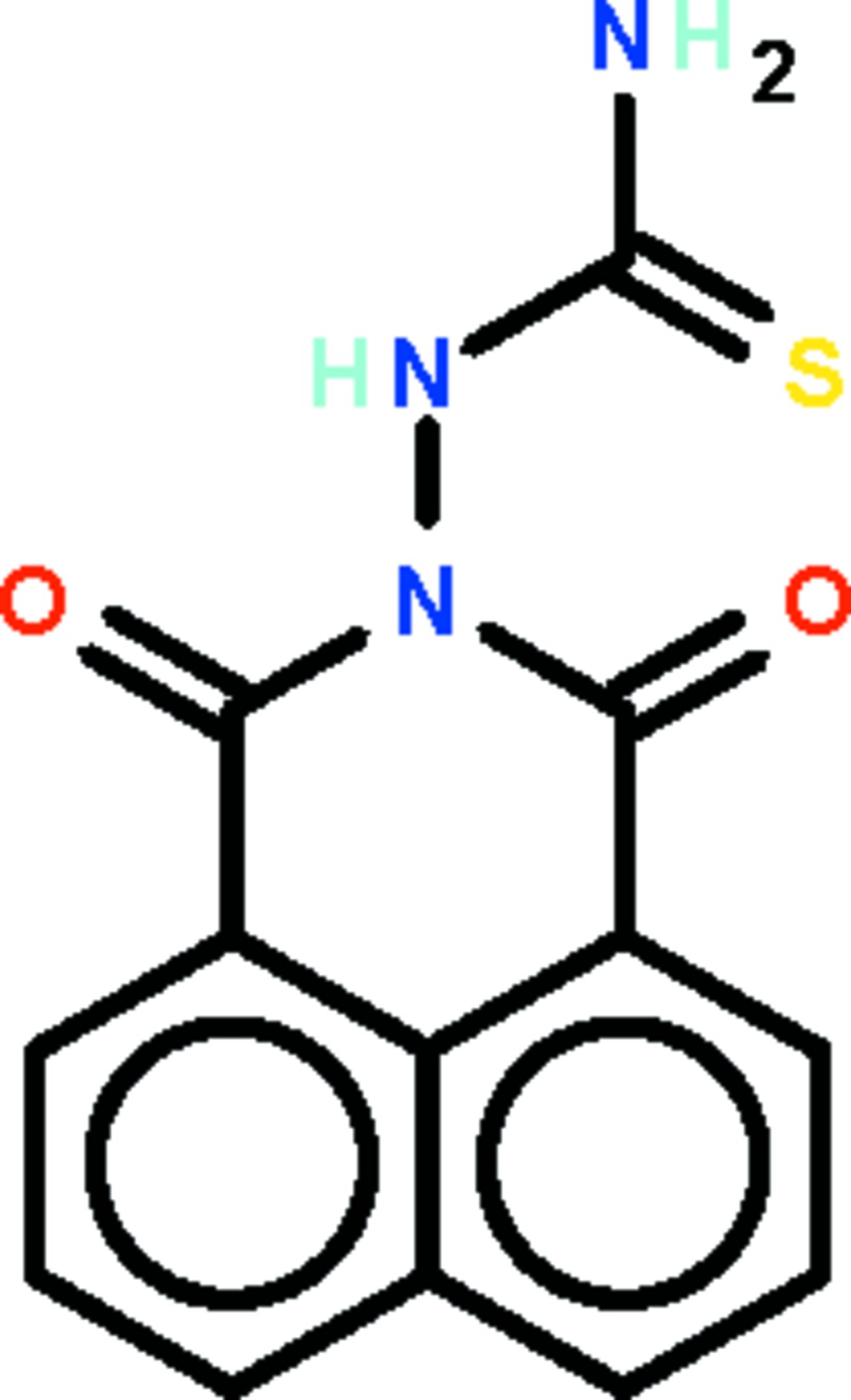


## Experimental

### 

#### Crystal data

C_13_H_9_N_3_O_2_S
*M* *_r_* = 271.29
Triclinic, 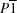

*a* = 4.5861 (2) Å
*b* = 11.0475 (4) Å
*c* = 22.5594 (9) Å
α = 89.196 (3)°
β = 88.073 (3)°
γ = 81.128 (3)°
*V* = 1128.61 (8) Å^3^
*Z* = 4
Cu *K*α radiation
μ = 2.58 mm^−1^
*T* = 100 K
0.25 × 0.10 × 0.03 mm

#### Data collection

Agilent SuperNova Dual diffractometer with an Atlas detector
Absorption correction: multi-scan (*CrysAlis PRO*; Agilent, 2012 ▶) *T*_min_ = 0.565, *T*_max_ = 0.927
14869 measured reflections
7974 independent reflections
7060 reflections with *I* > 2σ(*I*)
*R*_int_ = 0.076

#### Refinement

*R*[*F* ^2^ > 2σ(*F* ^2^)] = 0.065
*wR*(*F* ^2^) = 0.196
*S* = 1.11
7974 reflections
345 parameters
H-atom parameters constrained
Δρ_max_ = 0.47 e Å^−3^
Δρ_min_ = −0.73 e Å^−3^

### 

Data collection: *CrysAlis PRO* (Agilent, 2012 ▶); cell refinement: *CrysAlis PRO*; data reduction: *CrysAlis PRO*; program(s) used to solve structure: *SHELXS97* (Sheldrick, 2008 ▶); program(s) used to refine structure: *SHELXL97* (Sheldrick, 2008 ▶); molecular graphics: *X-SEED* (Barbour, 2001 ▶); software used to prepare material for publication: *publCIF* (Westrip, 2010 ▶).

## Supplementary Material

Crystal structure: contains datablock(s) global, I. DOI: 10.1107/S1600536812021812/bt5920sup1.cif

Structure factors: contains datablock(s) I. DOI: 10.1107/S1600536812021812/bt5920Isup2.hkl

Supplementary material file. DOI: 10.1107/S1600536812021812/bt5920Isup3.cml

Additional supplementary materials:  crystallographic information; 3D view; checkCIF report

## Figures and Tables

**Table 1:** Hydrogen-bond geometry (Å, °)

*D*—H⋯*A*	*D*—H	H⋯*A*	*D*⋯*A*	*D*—H⋯*A*
N2—H2⋯O1^i^	0.88	2.35	3.074 (3)	140
N2—H2⋯O1^ii^	0.88	2.33	2.993 (3)	132
N3—H31⋯O2^iii^	0.88	2.09	2.912 (3)	154
N5—H5⋯O3^iii^	0.88	2.55	3.301 (3)	144
N5—H5⋯O3^iv^	0.88	2.52	2.988 (3)	114
N6—H61⋯O4^i^	0.88	2.21	3.015 (3)	152

Symmetry codes: (i) 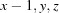; (ii) 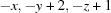; (iii) 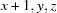; (iv) 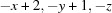.

**Fig. 1.**
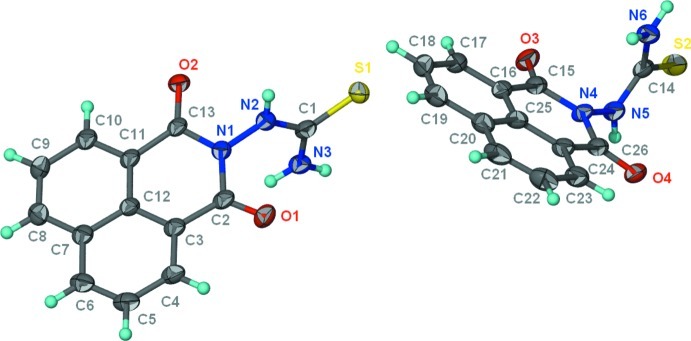
Anisotropic displacement ellipsoid plot (Barbour, 2001) of the two independent molecules of C13H9N3O2S at the 70% probability level; hydrogen atoms are drawn as spheres of arbitrary radius.

## supplementary crystallographic information

### Comment

1-(1,3-Dioxo-1,3-dihydro-2*H*-isoindol-2-yl)thiourea is a powerful antitumor drug that is synthesized from phthalic anhydride and thiosemicarbazide (Pessoa *et al.*, 2010). The title compound (Scheme I) is synthesized by using naphthalic acid anhydride in place of phthalic anhydride.

The crystal structure features two independent molecules. The azatricyclo-trideca-pentaene fused-ring is flat; the urea unit connected to the fused-ring is approximately perpendicular [dihedral angle 82.4 (1), 82.7 (1) °] (Fig. 1). Each independent molecule associates by an N–H···O hydrogen bond into a dimer; adjacent dimers are linked by N–H···O hydrogen bonds to form a chain running along the *a*-axis of the triclinic unit cell (Table 1).

### Experimental

Naphthalic acid anhydride (2.2 mmol, 0.44 g) was dissolved in glacial acetic acid (15 ml). A solution of thiosemicarbazide (2.8 mmol, 0.26 g) in glacial acetic acid (10 ml) was added. A solid formed immediately. The solid was collected, washed with water and ether. The compound was recrystallized from a mixture of toluene and DMF to yield colorless crystals.

### Refinement

Hydrogen atoms were placed in calculated positions [C–H 0.95, N–H 0.88 Å, *U*_iso_(H) 1.2*U*_eq_(C,*N*)] and were included in the refinement in the riding model approximation.

The crystal is a non-merohedral twin with a minor compound being of 49.6 (1)%.

The somewhat large weighting scheme is probably an artifact of twinning.

### Crystal data

**Table:**

C_13_H_9_N_3_O_2_S	*Z* = 4
*M**_r_* = 271.29	*F*(000) = 560
Triclinic, *P*1	*D*_x_ = 1.597 Mg m^−^^3^
Hall symbol: -P 1	Cu *K*α radiation, λ = 1.54184 Å
*a* = 4.5861 (2) Å	Cell parameters from 6016 reflections
*b* = 11.0475 (4) Å	θ = 3.9–76.4°
*c* = 22.5594 (9) Å	µ = 2.58 mm^−^^1^
α = 89.196 (3)°	*T* = 100 K
β = 88.073 (3)°	Plate, colorless
γ = 81.128 (3)°	0.25 × 0.10 × 0.03 mm
*V* = 1128.61 (8) Å^3^	

### Data collection

**Table:**

Agilent SuperNova Dual diffractometer with an Atlas detector	7974 independent reflections
Radiation source: SuperNova (Cu) X-ray Source	7060 reflections with *I* > 2σ(*I*)
Mirror monochromator	*R*_int_ = 0.076
Detector resolution: 10.4041 pixels mm^-1^	θ_max_ = 77.5°, θ_min_ = 3.9°
ω scan	*h* = −5→5
Absorption correction: multi-scan (*CrysAlis PRO*; Agilent, 2012)	*k* = −13→13
*T*_min_ = 0.565, *T*_max_ = 0.927	*l* = −28→24
14869 measured reflections	

### Refinement

**Table:**

Refinement on *F*^2^	Secondary atom site location: difference Fourier map
Least-squares matrix: full	Hydrogen site location: inferred from neighbouring sites
*R*[*F*^2^ > 2σ(*F*^2^)] = 0.065	H-atom parameters constrained
*wR*(*F*^2^) = 0.196	*w* = 1/[σ^2^(*F*_o_^2^) + (0.1312*P*)^2^ + 0.6561*P*] where *P* = (*F*_o_^2^ + 2*F*_c_^2^)/3
*S* = 1.11	(Δ/σ)_max_ = 0.001
7974 reflections	Δρ_max_ = 0.47 e Å^−^^3^
345 parameters	Δρ_min_ = −0.73 e Å^−^^3^
0 restraints	Extinction correction: *SHELXL97* (Sheldrick, 2008), Fc^*^=kFc[1+0.001xFc^2^λ^3^/sin(2θ)]^-1/4^
Primary atom site location: structure-invariant direct methods	Extinction coefficient: 0.007 (1)

### Fractional atomic coordinates and isotropic or equivalent isotropic displacement parameters (Å^2^)

**Table:**

	*x*	*y*	*z*	*U*_iso_*/*U*_eq_	
S1	−0.00401 (14)	0.90107 (6)	0.35711 (3)	0.02668 (18)	
S2	1.03964 (15)	0.64485 (7)	−0.15038 (3)	0.03115 (19)	
O1	0.3711 (4)	0.91572 (19)	0.53190 (8)	0.0280 (4)	
O2	−0.2323 (4)	0.63074 (18)	0.50792 (8)	0.0247 (4)	
O3	0.6884 (5)	0.5699 (2)	0.02003 (9)	0.0315 (4)	
O4	1.2207 (4)	0.88347 (18)	0.01639 (9)	0.0268 (4)	
N1	0.0726 (5)	0.7727 (2)	0.52057 (9)	0.0235 (4)	
N2	−0.0298 (5)	0.8305 (2)	0.46861 (10)	0.0243 (5)	
H2	−0.2080	0.8736	0.4689	0.029*	
N3	0.3931 (5)	0.7492 (2)	0.41554 (10)	0.0255 (5)	
H31	0.4532	0.7066	0.4473	0.031*	
H32	0.5037	0.7429	0.3828	0.031*	
N4	0.9302 (5)	0.7334 (2)	0.01767 (9)	0.0231 (4)	
N5	1.0573 (5)	0.6935 (2)	−0.03728 (10)	0.0248 (5)	
H5	1.2422	0.6574	−0.0398	0.030*	
N6	0.6345 (5)	0.7835 (2)	−0.08405 (10)	0.0288 (5)	
H61	0.5714	0.8196	−0.0505	0.035*	
H62	0.5260	0.7957	−0.1156	0.035*	
C1	0.1363 (6)	0.8228 (2)	0.41709 (11)	0.0231 (5)	
C2	0.2727 (5)	0.8289 (3)	0.55335 (11)	0.0237 (5)	
C3	0.3503 (5)	0.7740 (2)	0.61137 (11)	0.0228 (5)	
C4	0.5301 (6)	0.8282 (3)	0.64730 (12)	0.0257 (5)	
H4	0.6002	0.9012	0.6347	0.031*	
C5	0.6094 (6)	0.7749 (3)	0.70274 (12)	0.0288 (6)	
H5A	0.7324	0.8127	0.7274	0.035*	
C6	0.5111 (6)	0.6693 (3)	0.72147 (12)	0.0277 (6)	
H6	0.5666	0.6347	0.7590	0.033*	
C7	0.3271 (5)	0.6110 (3)	0.68538 (11)	0.0241 (5)	
C8	0.2256 (6)	0.5010 (3)	0.70251 (12)	0.0264 (5)	
H8	0.2803	0.4639	0.7396	0.032*	
C9	0.0490 (6)	0.4467 (3)	0.66639 (12)	0.0258 (5)	
H9	−0.0141	0.3718	0.6783	0.031*	
C10	−0.0390 (6)	0.5017 (3)	0.61165 (12)	0.0255 (5)	
H10	−0.1647	0.4649	0.5872	0.031*	
C11	0.0577 (5)	0.6086 (2)	0.59394 (11)	0.0222 (5)	
C12	0.2453 (5)	0.6650 (2)	0.62956 (11)	0.0224 (5)	
C13	−0.0487 (5)	0.6671 (2)	0.53792 (11)	0.0222 (5)	
C14	0.8931 (6)	0.7108 (3)	−0.08703 (12)	0.0254 (5)	
C15	0.7413 (6)	0.6591 (3)	0.04568 (12)	0.0254 (5)	
C16	0.6217 (6)	0.6980 (3)	0.10479 (12)	0.0249 (5)	
C17	0.4327 (6)	0.6302 (3)	0.13435 (13)	0.0294 (6)	
H17	0.3849	0.5583	0.1169	0.035*	
C18	0.3116 (6)	0.6675 (3)	0.19013 (13)	0.0330 (6)	
H18	0.1787	0.6215	0.2100	0.040*	
C19	0.3827 (6)	0.7698 (3)	0.21630 (13)	0.0325 (6)	
H19	0.3023	0.7928	0.2546	0.039*	
C20	0.5755 (6)	0.8421 (3)	0.18675 (12)	0.0286 (6)	
C21	0.6513 (6)	0.9491 (3)	0.21190 (13)	0.0328 (6)	
H21	0.5746	0.9737	0.2502	0.039*	
C22	0.8338 (7)	1.0178 (3)	0.18183 (14)	0.0341 (6)	
H22	0.8777	1.0907	0.1990	0.041*	
C23	0.9566 (6)	0.9808 (3)	0.12557 (12)	0.0295 (6)	
H23	1.0853	1.0281	0.1052	0.035*	
C24	0.8896 (6)	0.8757 (3)	0.10003 (12)	0.0249 (5)	
C25	0.6951 (6)	0.8056 (3)	0.12967 (12)	0.0258 (5)	
C26	1.0303 (6)	0.8364 (3)	0.04201 (12)	0.0243 (5)	

### Atomic displacement parameters (Å^2^)

**Table:**

	*U*^11^	*U*^22^	*U*^33^	*U*^12^	*U*^13^	*U*^23^
S1	0.0238 (3)	0.0370 (3)	0.0193 (3)	−0.0052 (2)	0.0006 (2)	0.0020 (2)
S2	0.0249 (3)	0.0490 (4)	0.0204 (3)	−0.0084 (3)	0.0019 (2)	−0.0066 (3)
O1	0.0271 (9)	0.0336 (10)	0.0251 (10)	−0.0113 (8)	0.0060 (7)	−0.0019 (8)
O2	0.0200 (9)	0.0343 (10)	0.0213 (9)	−0.0089 (7)	−0.0012 (7)	−0.0016 (7)
O3	0.0315 (10)	0.0392 (11)	0.0280 (10)	−0.0190 (8)	0.0005 (8)	−0.0043 (8)
O4	0.0201 (8)	0.0343 (10)	0.0275 (9)	−0.0092 (7)	0.0010 (7)	−0.0001 (8)
N1	0.0203 (10)	0.0334 (11)	0.0179 (10)	−0.0078 (9)	0.0001 (8)	0.0010 (9)
N2	0.0177 (10)	0.0338 (11)	0.0210 (10)	−0.0037 (8)	0.0010 (8)	0.0030 (9)
N3	0.0182 (10)	0.0368 (12)	0.0216 (11)	−0.0051 (9)	0.0017 (8)	−0.0020 (9)
N4	0.0168 (9)	0.0354 (11)	0.0184 (10)	−0.0090 (8)	0.0036 (8)	−0.0053 (9)
N5	0.0195 (10)	0.0359 (12)	0.0195 (10)	−0.0066 (8)	0.0028 (8)	−0.0043 (9)
N6	0.0195 (10)	0.0457 (13)	0.0212 (11)	−0.0053 (9)	0.0004 (8)	−0.0001 (10)
C1	0.0198 (11)	0.0286 (12)	0.0227 (12)	−0.0097 (9)	0.0007 (9)	−0.0034 (10)
C2	0.0172 (11)	0.0340 (13)	0.0209 (12)	−0.0079 (10)	0.0050 (9)	−0.0040 (10)
C3	0.0169 (11)	0.0310 (12)	0.0207 (12)	−0.0053 (9)	0.0029 (9)	−0.0031 (10)
C4	0.0193 (11)	0.0338 (13)	0.0254 (13)	−0.0083 (10)	0.0019 (10)	−0.0050 (10)
C5	0.0202 (12)	0.0404 (15)	0.0266 (13)	−0.0065 (11)	−0.0012 (10)	−0.0081 (11)
C6	0.0189 (12)	0.0444 (16)	0.0199 (12)	−0.0047 (11)	0.0004 (9)	−0.0052 (11)
C7	0.0184 (11)	0.0326 (13)	0.0212 (12)	−0.0044 (10)	0.0029 (9)	−0.0014 (10)
C8	0.0223 (12)	0.0344 (13)	0.0212 (12)	−0.0014 (10)	0.0038 (10)	0.0021 (10)
C9	0.0259 (12)	0.0283 (12)	0.0236 (12)	−0.0066 (10)	0.0052 (10)	0.0013 (10)
C10	0.0223 (12)	0.0342 (13)	0.0210 (12)	−0.0086 (10)	0.0034 (9)	−0.0025 (10)
C11	0.0181 (11)	0.0306 (12)	0.0188 (11)	−0.0078 (9)	0.0040 (9)	−0.0017 (10)
C12	0.0174 (11)	0.0303 (12)	0.0204 (12)	−0.0066 (9)	0.0009 (9)	−0.0003 (10)
C13	0.0163 (11)	0.0310 (12)	0.0201 (12)	−0.0071 (9)	0.0042 (9)	−0.0010 (10)
C14	0.0197 (12)	0.0358 (13)	0.0229 (12)	−0.0121 (10)	0.0015 (9)	−0.0007 (10)
C15	0.0201 (11)	0.0351 (13)	0.0224 (13)	−0.0083 (10)	−0.0025 (10)	−0.0006 (10)
C16	0.0183 (11)	0.0365 (13)	0.0208 (12)	−0.0074 (10)	−0.0006 (9)	0.0018 (10)
C17	0.0211 (12)	0.0403 (15)	0.0276 (14)	−0.0080 (11)	−0.0008 (10)	0.0057 (11)
C18	0.0205 (12)	0.0484 (17)	0.0293 (14)	−0.0048 (11)	0.0031 (10)	0.0120 (12)
C19	0.0228 (13)	0.0507 (17)	0.0218 (13)	0.0007 (12)	0.0019 (10)	0.0040 (12)
C20	0.0216 (12)	0.0405 (15)	0.0220 (13)	0.0002 (11)	−0.0009 (10)	0.0007 (11)
C21	0.0269 (13)	0.0474 (17)	0.0217 (13)	0.0027 (12)	−0.0036 (10)	−0.0064 (12)
C22	0.0346 (15)	0.0380 (15)	0.0288 (14)	−0.0008 (12)	−0.0082 (12)	−0.0079 (12)
C23	0.0271 (13)	0.0381 (15)	0.0242 (13)	−0.0069 (11)	−0.0036 (11)	−0.0028 (11)
C24	0.0213 (12)	0.0332 (13)	0.0212 (12)	−0.0068 (10)	−0.0016 (10)	−0.0035 (10)
C25	0.0174 (11)	0.0388 (14)	0.0214 (12)	−0.0045 (10)	−0.0017 (9)	−0.0006 (11)
C26	0.0189 (11)	0.0344 (13)	0.0207 (12)	−0.0074 (10)	−0.0019 (9)	−0.0010 (10)

### Geometric parameters (Å, º)

**Table:**

S1—C1	1.687 (3)	C6—H6	0.9500
S2—C14	1.685 (3)	C7—C8	1.411 (4)
O1—C2	1.210 (3)	C7—C12	1.423 (4)
O2—C13	1.216 (3)	C8—C9	1.374 (4)
O3—C15	1.210 (3)	C8—H8	0.9500
O4—C26	1.210 (3)	C9—C10	1.412 (4)
N1—N2	1.388 (3)	C9—H9	0.9500
N1—C13	1.413 (3)	C10—C11	1.374 (4)
N1—C2	1.417 (3)	C10—H10	0.9500
N2—C1	1.364 (3)	C11—C12	1.414 (3)
N2—H2	0.8800	C11—C13	1.475 (4)
N3—C1	1.324 (3)	C15—C16	1.469 (4)
N3—H31	0.8800	C16—C17	1.380 (4)
N3—H32	0.8800	C16—C25	1.413 (4)
N4—N5	1.399 (3)	C17—C18	1.400 (4)
N4—C15	1.412 (3)	C17—H17	0.9500
N4—C26	1.414 (3)	C18—C19	1.370 (5)
N5—C14	1.367 (3)	C18—H18	0.9500
N5—H5	0.8800	C19—C20	1.423 (4)
N6—C14	1.326 (4)	C19—H19	0.9500
N6—H61	0.8800	C20—C21	1.414 (4)
N6—H62	0.8800	C20—C25	1.422 (4)
C2—C3	1.466 (4)	C21—C22	1.372 (5)
C3—C4	1.381 (3)	C21—H21	0.9500
C3—C12	1.415 (4)	C22—C23	1.411 (4)
C4—C5	1.409 (4)	C22—H22	0.9500
C4—H4	0.9500	C23—C24	1.384 (4)
C5—C6	1.371 (4)	C23—H23	0.9500
C5—H5A	0.9500	C24—C25	1.415 (4)
C6—C7	1.421 (4)	C24—C26	1.480 (4)
			
N2—N1—C13	116.5 (2)	C10—C11—C12	121.2 (2)
N2—N1—C2	117.5 (2)	C10—C11—C13	118.6 (2)
C13—N1—C2	125.9 (2)	C12—C11—C13	120.1 (2)
C1—N2—N1	122.3 (2)	C11—C12—C3	121.7 (2)
C1—N2—H2	118.9	C11—C12—C7	118.8 (2)
N1—N2—H2	118.9	C3—C12—C7	119.4 (2)
C1—N3—H31	120.0	O2—C13—N1	120.4 (2)
C1—N3—H32	120.0	O2—C13—C11	123.9 (2)
H31—N3—H32	120.0	N1—C13—C11	115.7 (2)
N5—N4—C15	116.5 (2)	N6—C14—N5	118.8 (2)
N5—N4—C26	116.2 (2)	N6—C14—S2	123.1 (2)
C15—N4—C26	126.9 (2)	N5—C14—S2	118.0 (2)
C14—N5—N4	119.8 (2)	O3—C15—N4	118.9 (2)
C14—N5—H5	120.1	O3—C15—C16	124.9 (3)
N4—N5—H5	120.1	N4—C15—C16	116.2 (2)
C14—N6—H61	120.0	C17—C16—C25	121.2 (3)
C14—N6—H62	120.0	C17—C16—C15	119.2 (3)
H61—N6—H62	120.0	C25—C16—C15	119.7 (2)
N3—C1—N2	118.5 (2)	C16—C17—C18	119.9 (3)
N3—C1—S1	122.9 (2)	C16—C17—H17	120.1
N2—C1—S1	118.5 (2)	C18—C17—H17	120.1
O1—C2—N1	119.1 (2)	C19—C18—C17	120.7 (3)
O1—C2—C3	124.6 (2)	C19—C18—H18	119.7
N1—C2—C3	116.3 (2)	C17—C18—H18	119.7
C4—C3—C12	120.6 (2)	C18—C19—C20	120.8 (3)
C4—C3—C2	119.5 (2)	C18—C19—H19	119.6
C12—C3—C2	119.9 (2)	C20—C19—H19	119.6
C3—C4—C5	119.9 (3)	C21—C20—C25	118.8 (3)
C3—C4—H4	120.1	C21—C20—C19	122.5 (3)
C5—C4—H4	120.1	C25—C20—C19	118.7 (3)
C6—C5—C4	120.8 (2)	C22—C21—C20	121.1 (3)
C6—C5—H5A	119.6	C22—C21—H21	119.5
C4—C5—H5A	119.6	C20—C21—H21	119.5
C5—C6—C7	120.7 (3)	C21—C22—C23	120.3 (3)
C5—C6—H6	119.6	C21—C22—H22	119.8
C7—C6—H6	119.6	C23—C22—H22	119.8
C8—C7—C6	122.5 (2)	C24—C23—C22	120.0 (3)
C8—C7—C12	118.9 (2)	C24—C23—H23	120.0
C6—C7—C12	118.6 (2)	C22—C23—H23	120.0
C9—C8—C7	121.0 (2)	C23—C24—C25	120.4 (2)
C9—C8—H8	119.5	C23—C24—C26	119.0 (2)
C7—C8—H8	119.5	C25—C24—C26	120.6 (2)
C8—C9—C10	120.3 (2)	C16—C25—C24	121.8 (2)
C8—C9—H9	119.9	C16—C25—C20	118.8 (3)
C10—C9—H9	119.9	C24—C25—C20	119.4 (3)
C11—C10—C9	119.8 (2)	O4—C26—N4	120.4 (2)
C11—C10—H10	120.1	O4—C26—C24	125.1 (2)
C9—C10—H10	120.1	N4—C26—C24	114.5 (2)
			
C13—N1—N2—C1	−105.9 (3)	C10—C11—C13—N1	−176.4 (2)
C2—N1—N2—C1	78.2 (3)	C12—C11—C13—N1	6.3 (3)
C15—N4—N5—C14	77.9 (3)	N4—N5—C14—N6	11.6 (4)
C26—N4—N5—C14	−108.8 (3)	N4—N5—C14—S2	−171.95 (18)
N1—N2—C1—N3	5.9 (4)	N5—N4—C15—O3	−3.5 (4)
N1—N2—C1—S1	−177.69 (19)	C26—N4—C15—O3	−176.0 (2)
N2—N1—C2—O1	−8.2 (4)	N5—N4—C15—C16	176.8 (2)
C13—N1—C2—O1	176.4 (2)	C26—N4—C15—C16	4.3 (4)
N2—N1—C2—C3	173.1 (2)	O3—C15—C16—C17	−0.5 (4)
C13—N1—C2—C3	−2.4 (4)	N4—C15—C16—C17	179.2 (2)
O1—C2—C3—C4	5.2 (4)	O3—C15—C16—C25	−178.7 (3)
N1—C2—C3—C4	−176.2 (2)	N4—C15—C16—C25	1.0 (4)
O1—C2—C3—C12	−173.6 (2)	C25—C16—C17—C18	−0.3 (4)
N1—C2—C3—C12	5.1 (4)	C15—C16—C17—C18	−178.6 (3)
C12—C3—C4—C5	−0.3 (4)	C16—C17—C18—C19	−1.1 (4)
C2—C3—C4—C5	−179.0 (2)	C17—C18—C19—C20	1.5 (4)
C3—C4—C5—C6	0.3 (4)	C18—C19—C20—C21	179.2 (3)
C4—C5—C6—C7	0.1 (4)	C18—C19—C20—C25	−0.5 (4)
C5—C6—C7—C8	178.8 (2)	C25—C20—C21—C22	0.8 (4)
C5—C6—C7—C12	−0.5 (4)	C19—C20—C21—C22	−178.9 (3)
C6—C7—C8—C9	−179.7 (2)	C20—C21—C22—C23	−1.8 (5)
C12—C7—C8—C9	−0.4 (4)	C21—C22—C23—C24	1.0 (4)
C7—C8—C9—C10	−1.2 (4)	C22—C23—C24—C25	0.8 (4)
C8—C9—C10—C11	1.4 (4)	C22—C23—C24—C26	−177.7 (2)
C9—C10—C11—C12	0.1 (4)	C17—C16—C25—C24	179.7 (3)
C9—C10—C11—C13	−177.2 (2)	C15—C16—C25—C24	−2.1 (4)
C10—C11—C12—C3	179.0 (2)	C17—C16—C25—C20	1.4 (4)
C13—C11—C12—C3	−3.8 (4)	C15—C16—C25—C20	179.6 (2)
C10—C11—C12—C7	−1.7 (4)	C23—C24—C25—C16	179.9 (3)
C13—C11—C12—C7	175.5 (2)	C26—C24—C25—C16	−1.6 (4)
C4—C3—C12—C11	179.1 (2)	C23—C24—C25—C20	−1.8 (4)
C2—C3—C12—C11	−2.1 (4)	C26—C24—C25—C20	176.7 (2)
C4—C3—C12—C7	−0.2 (4)	C21—C20—C25—C16	179.4 (2)
C2—C3—C12—C7	178.6 (2)	C19—C20—C25—C16	−0.9 (4)
C8—C7—C12—C11	1.9 (4)	C21—C20—C25—C24	1.0 (4)
C6—C7—C12—C11	−178.8 (2)	C19—C20—C25—C24	−179.3 (3)
C8—C7—C12—C3	−178.8 (2)	N5—N4—C26—O4	−1.8 (4)
C6—C7—C12—C3	0.5 (4)	C15—N4—C26—O4	170.8 (3)
N2—N1—C13—O2	0.2 (4)	N5—N4—C26—C24	179.8 (2)
C2—N1—C13—O2	175.7 (2)	C15—N4—C26—C24	−7.7 (4)
N2—N1—C13—C11	−178.7 (2)	C23—C24—C26—O4	6.3 (4)
C2—N1—C13—C11	−3.2 (4)	C25—C24—C26—O4	−172.3 (3)
C10—C11—C13—O2	4.8 (4)	C23—C24—C26—N4	−175.4 (2)
C12—C11—C13—O2	−172.6 (2)	C25—C24—C26—N4	6.1 (4)

### Hydrogen-bond geometry (Å, º)

**Table:**

*D*—H···*A*	*D*—H	H···*A*	*D*···*A*	*D*—H···*A*
N2—H2···O1^i^	0.88	2.35	3.074 (3)	140
N2—H2···O1^ii^	0.88	2.33	2.993 (3)	132
N3—H31···O2^iii^	0.88	2.09	2.912 (3)	154
N5—H5···O3^iii^	0.88	2.55	3.301 (3)	144
N5—H5···O3^iv^	0.88	2.52	2.988 (3)	114
N6—H61···O4^i^	0.88	2.21	3.015 (3)	152

Symmetry codes: (i) *x*−1, *y*, *z*; (ii) −*x*, −*y*+2, −*z*+1; (iii) *x*+1, *y*, *z*; (iv) −*x*+2, −*y*+1, −*z*.
